# Evaluation of Zebrafish Toxicology and Biomedical Potential of *Aeromonas hydrophila* Mediated Copper Sulfide Nanoparticles

**DOI:** 10.1155/2022/7969825

**Published:** 2022-01-28

**Authors:** S. Rajeshkumar, J. Santhoshkumar, M. Vanaja, P. Sivaperumal, M. Ponnanikajamideen, Daoud Ali, Kalirajan Arunachalam

**Affiliations:** ^1^Centre for Transdisciplinary Research, Nanobiomedicine Lab, Department of Pharmacology, Saveetha Dental College and Hospital, SIMATS, Chennai 600077, India; ^2^Department of Biotechnology, Saveetha School of Engineering, Saveetha Institute of Medical and Technical Science (SIMATS), 602105, Chennai, India; ^3^SPKCES, Manonmaniam Sundaranar University, Alwarkurichi, 627410 Tamil Nadu, India; ^4^Department of Pharmacology and Toxicology, University of Mississippi Medical Centre, Jackson, Mississippi, USA; ^5^Department of Zoology, College of Science, King Saud University, PO Box 2455, Riyadh 11451, Saudi Arabia; ^6^Department of Science and Mathematics, School of Science, Engineering and Technology, Mulungushi University, Kabwe 80415, Zambia

## Abstract

The present study deals with extracellular synthesis and characterization of copper sulfide (CuS) nanoparticles using *Aeromonas hydrophila*, and the biological applications of the synthesized CuS like antibacterial, anti-inflammatory, and antioxidant activity were reported. Further, the toxicological effects of the CuS were evaluated using zebrafish as an animal model. The primary step of the synthesis was carried out by adding the precursor copper sulfates to the culture supernatant of *Aeromonas hydrophila*. The UV-visible spectrophotometer was used to characterize the synthesized nanoparticles, and the peak was obtained at 307 nm through the reduction process. Fourier transform infrared spectroscopy (FTIR) was involved to find out the functional groups (carboxylic acid, alcohols, alkanes, and nitro compounds) associated with copper sulfide nanoparticles (CuS-NPs). Atomic force microscopy (AFM) was used to characterize the CuS topographically, and a scanning electron microscope (SEM) revealed about 200 nm sized CuS nanoparticles with agglomerated structures. Overall, the characterized nanoparticles can be considered as a potential candidate with therapeutic proficiencies as antibacterial, antioxidant, and anti-inflammatory mediator/agents.

## 1. Introduction

Copper is a common element that exists naturally in the environment and distributes through anthropogenic activities. It is soft, flexible metal with high thermal and electrical conductivity. Like iron, copper is a trace element required for the formation of body tissues and red blood cells. The economic value of combining crystalline copper and semiconductor nanoparticles is used in various fields including catalysis, material science, solar cells, the environment aspects, and medicine, owing to their unique properties [1, 2]. The size of semiconductor nanoparticles reduced into nanometer scale is due to quantum effects. As a result, size, shape, and quantum effects of such reduction play a critical role in determining the properties of semiconductor nanoparticles. Copper sulfide nanoparticles have tremendous applications, such as in solar cells [3–5], wastewater treatment, and sensors [6, 7], and also used in the manufacturing of other electronic components [8]. Different techniques, like physical, chemical, and biological methods, are used to synthesize copper sulfide nanoparticles [9, 10]. The biological method has several advantages such as environmentally friendly, cost-effective, and usage of less toxic chemicals in the synthesis process [11]. The described biological method used the biological sources such as bacteria [12], fungi [13–15], algae, plants [16, 17], and other biological materials. Here, the bacteria is used for nanoparticle synthesis, as it produces metal resistance and participates in effectively reducing the ions into nanoparticles. When bacteria were exposed to low metal ion concentrations, they develop resistance and induce nanoparticle synthesis [18]. Bacterial synthesis of nanoparticles can be done either (i) intracellular or extracellular. Both methods are used to obtain the controlled size and shape of the nanoparticle during synthesis process. Bacterial culture supernatant was used for the extracellular synthesis of nanoparticles, and this method can be reproduced for the better size and shape with controllable synthesis of nanoparticles than the intracellular one [19]. There are plenty of reports on the synthesis of copper nanoparticles using bacteria such as acidophilic sulfate-reducing bacteria [20], *Escherichia coli* [21], *Pseudomonas* sp. [22], *Pseudomonas stutzeri* [23, 24], *Serratia sp.* [25], *Streptomyces sp.* [26], *Morganella* bacteria [27], *Pseudomonas fluorescens* [28], and *Shewanella oneidensis* MR-1 [12]; among them, extracellular synthesis of copper sulfide nanoparticles (CuS-NPs) using the bacteria *Aeromonas hydrophila* has rarely been reported. The synthesized nanoparticles were characterized using UV-vis spectra, XRD, FTIR, AFM, SEM, and EDX analyses; biological applications such as antibacterial, antioxidant, and anti-inflammatory properties of the CuS-NPs were assessed to determine their biological role. In addition, characterized nanoparticles and their biological roles were assessed by antibacterial, anti-inflammatory, and antioxidant activities. Furthermore, a toxicological assay of CuS-NPs was performed in zebrafish embryos as an animal model.

## 2. Materials and Methods

### 2.1. Materials Used

The bacterial strain *Aeromonas hydrophila* (7966) was purchased from the American Type Culture Collection, Tamil Nadu, India. Nutrient broth, Mueller-Hinton agar, DPPH, and media were purchased from Sigma-Aldrich, India, and the standard antibiotics were purchased from Hi-Media Laboratories, Mumbai, India. In addition, zebrafish (*Danio rerio*) embryos were purchased from Tarun fish farm, Manimangalam, Chennai.

### 2.2. Culturing *Aeromonas hydrophila* and Synthesis of CuS Nanoparticles

*Aeromonas hydrophila* is a rod-shaped, gram-negative bacterium commonly found in brackish water and causes disease in fish. It is used in this experiment for the synthesis of copper sulfide nanoparticles. The bacterial culture was grown in the nutrient broth with the pH 7.2 and incubated at 30°C for 24 hours in a shaking incubator at 120 rpm. The culture was centrifuged at 10000 rpm for 10 min and collected the cell-free supernatant. The nanoparticle synthesis was attempted by following slightly modified protocol [29] and related applications as well. The bacterial supernatant was used for the extracellular synthesis of copper sulfide nanoparticles by adding the precursor material, 1 mM copper sulfates thoroughly mixed and incubated for reduction process. After the addition of copper sulfate, the reaction mixture turns into greenish-blue from greenish-brown color. The color change indicates the synthesis of CuS-NPs.

### 2.3. Characterization of Synthesized Copper Sulfide Nanoparticles

The crystalline character of the synthesized copper sulfide nanoparticles was investigated with the help of powder XRD (XRD D8 ADVANCE BRUKER) analysis. The X-ray patterns were obtained in the 2 theta configurations in the range of 20°–80°. After drying off the purified CuS nanoparticles, the sample's elemental composition was analyzed with energy dispersive analysis of X-ray spectroscopy (scanning electron microscope predicted ZEISS (EVD18)) and the morphology and size. Absorption spectra were determined by a UV-vis spectrophotometer (SHIMADZU UV-1280) with a frequency range from 300 nm to 320 nm.

## 3. Biomedical Applications

### 3.1. Antibacterial Activity of Copper Sulfide (CuS) Nanoparticles

Antibacterial activity was done by the agar well diffusion method using various pathogenic bacteria such as *Vibrio parahaemolyticus*, *Serratia marcescens*, *Proteus sp*, *E. coli*, and *Bacillus* sp. Fresh bacterial inoculum of pathogens was spread on sterile Mueller-Hinton agar plate using sterile cotton swabs, respectively. About four wells were made in each plate using a sterile gel puncture for adding different concentrations of CuS nanoparticles. Various concentrations (25 and 100 *μ*g/mL) of copper sulfide nanoparticles were incorporated into each well, and negative control was kept; a standard antibiotic (chloramphenicol) (25 *μ*g/mL) was used as positive control. All the plates were incubated at 35°C for 24 hours and observed zone formation around the well.

### 3.2. Antioxidant Activity

Antioxidant activity of biosynthesized CuS nanoparticles was performed by assaying the free radical scavenging effect on DPPH (2-diphenyl-2-picrylhydrazyl). For the present activity, 1 mL of different concentrations of CuS nanoparticles (25, 50, 75, and 100 *μ*g/mL) was mixed with 1 mL of 1 mM DPPH prepared using methanol. DPPH solution prepared in methanol without sample was considered as control. Then, the reaction solutions were mixed thoroughly by vortexing and incubated at room temperature under dark conditions for up to 30 min. After incubation, the discolorations of DPPH from purple to yellow were observed and the absorbance of the DPPH scavenging by nanoparticles was recorded at 517 nm using a UV-vis spectrophotometer.

The percentage of inhibition was calculated using the following formula:

(1) $$\begin{array}{c} \text{Inhibition }\%=\text{OD of control}-\text{OD of test sample}/\text{OD of control}*100. \end{array}$$

### 3.3. Anti-Inflammatory Activity

The inflammation inhibitory effects of CuS nanoparticles were performed by membrane stabilizing activity in human red blood cells (RBCs). Fresh human blood (10 mL) was collected and mixed with 10 mL of saline (pH 7.2) solution. The mixed saline blood was centrifuged at 3000 rpm for 10 mins and washed with saline solution. This process was repeated three times. Presently, 2 mL of CuS nanoparticles was taken in different concentrations (25, 50, 75, and 100 *μ*g/mL) and mixed with 1 mL of RBCs (10%*v*/*v*), respectively. The standard drug was prepared by mixing 2 mL of diclofenac sodium (25 mg) with 1 mL of RBCs in saline. Distilled water instead of saline is considered as a control. All the mixtures were incubated at 56°C for 30 mins. After incubation, all the tubes were cooled and centrifuged at 2500 rpm for 5 mins. The absorbance of the supernatants was read at 560 nm.

The percentage of inhibition was calculated using the following formula:

(2) $$\begin{array}{c} \text{Inhibition }\%=\text{OD of control}-\text{OD of test sample}/\text{OD of control}*100. \end{array}$$

### 3.4. Toxicology Analysis of Synthesized Copper Nanoparticles using Zebrafish

The embryos of Zebrafish were incubated at 26°C in culture water. Randomly selected embryos at 4 hours postfertilization (sphere stage) were maintained with 10 mL of zebrafish culture water. Healthy embryos were selected and placed in 96-well culture plates containing 0.2 mL of culture water. To each well, 0.1 mL of different concentrations of CuS nanoparticles (0 to 150 *μ*g/mL) was added, respectively. Three replicates were included, and the embryos in the culture medium were considered as control. Then, the plates were incubated at 26°C and observed the developmental status of the embryos and zebrafish larvae at different fertilizing periods. Hatching and mortality rates in percentages were calculated at every 12 h from the total number of survival embryos. Malfunction in embryos caused by nanoparticles was observed using a microscope.

### 3.5. Statistical Analyses

All the experiments were performed in triplicate, and the obtained data were expressed as mean values ± standard error (SE). Data were interpreted using GraphPad 6.1 software. Two-way ANOVA was performed using the Bonferroni post hoc test to evaluate the significant differences between groups (standard drug and nanomaterial sample). The significant level for different concentrations of standard drug and test samples was set top ≤ 0.05.

## 4. Results and Discussion

### 4.1. UV-Visible Spectroscopy

**Figure 1:**
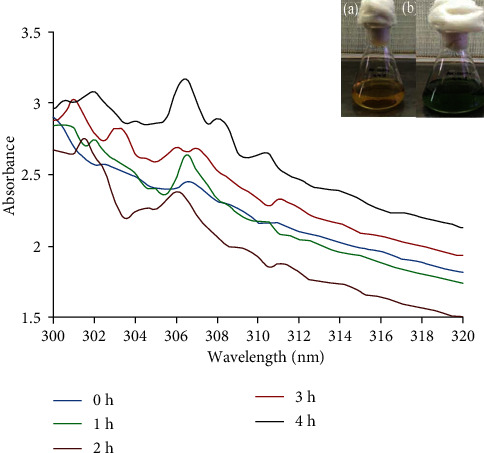
UV spectra of extracellularly synthesized copper sulfide nanoparticles recorded at various time intervals. Insert figure (“a” and “b”) shows color change of culture supernatant from yellow to green indicates synthesis of copper sulfide nanoparticles by extracellularly.

The optical properties and the bioreduction of nanoparticles have been studied using the UV-vis absorption spectrum. A color change from pale yellow to green was primarily observed when the CuSO_4_ was added to the cell-free supernatant. After incubation, the color of the reaction mixture changed into greenish-brown (Figure 1). Several strong peaks for copper sulfide nanoparticles were observed between 300 and 320 nm. As the size of the CuSO_4_ to the bulk decreases the absorption shifts to shorter wavelengths (blue shift), which were observed in the nanoregion, the maximum absorption spectrum that produced blue shift during synthesis was observed at 307 nm, which implies the presence of copper synthesized in high amounts.

### 4.2. Nature and Functional Group of CuS Nanoparticles

**Figure 2:**
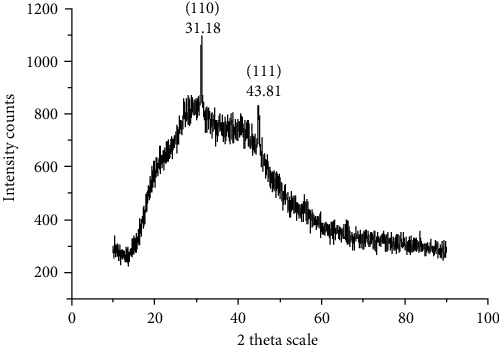
XRD shows crystalline nature of copper sulfide nanoparticles.

XRD result revealed the crystalline nature of the synthesized copper sulfide nanoparticles. The peaks obtained represent the presence of copper sulfide, and it is confirmed by the planes (110) and (111), which are corresponding to the 2*θ* degree 31.18° and 43.81°, respectively (Figure 2). The average size of nanoparticles was found to be 20 nm which was calculated using the formula Debye-Scherrer equations *D* = *Kλ*/(*β* Cos*θ*). Here, *D* is the particle size, *K* is the Blanks constant, *λ* is the X-ray wavelength, *β* is the FWHM intensity, and *θ* is the Bragg angle.

**Figure 3:**
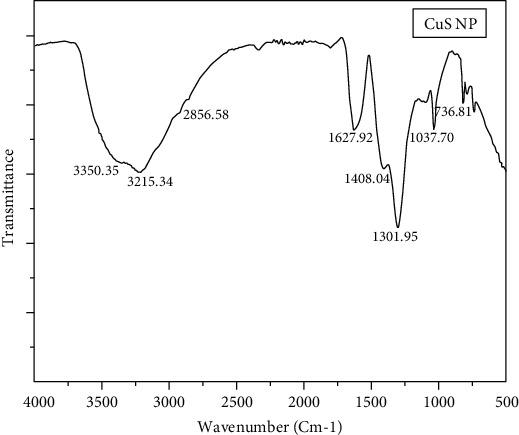
FTIR spectrum shows the functional molecules present in copper sulfide nanoparticles.

The functional groups present in the supernatant of *A. hydrophila* were revealed through the frequency peaks at the wavenumber of 3350.35 cm^−1^ pointed to the O-H stretching vibration of carboxylic acid. Likely, two small peaks were found at 3215.34 and 2856.58 cm^−1^ signifying the C-H stretching vibration of alkanes and represented to be asymmetric. Similarly, two peaks at 1627.92 and 1408.04 cm^−1^ were assigned to N–O asymmetric stretch nitro compounds. The two wavenumbers at 1301.95 and 1037.70 cm^−1^ were corresponding to the presence of C-O stretching alcohols, respectively. A frequency band at 736.81 cm^−1^ was due to the vibrations of the CH_3_, and the peak implies the weak bond with C-C skeleton vibration (Figure 3).

### 4.3. Surface and Shape of the CuS Nanoparticles

**Figure 4:**
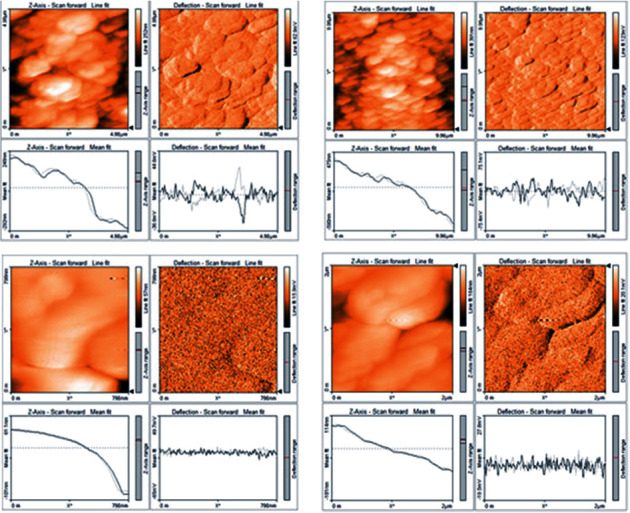
AFM image shows surface morphology of copper sulfide nanoparticles.

**Figure 5:**
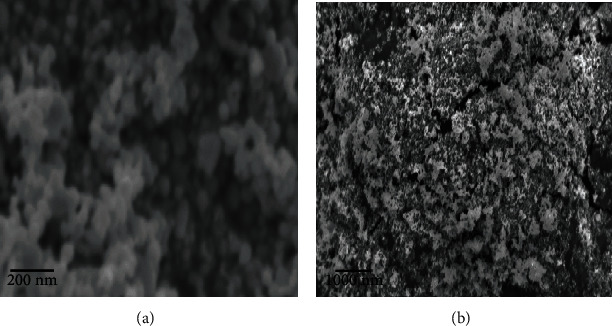
SEM analysis of copper sulfide nanoparticle synthesized by *Aeromonas hydrophila* scanned at different magnification ranges: (a) scale bar: 2 *μ*m; (b) scale bar: 200 nm.

**Figure 6:**
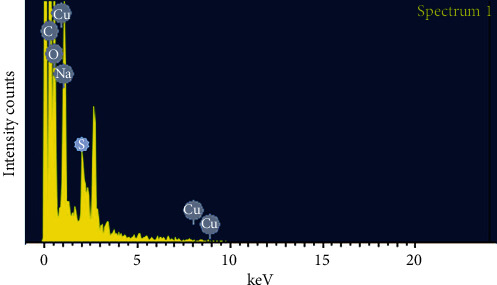
EDX spectrum of CuS nanoparticles.

The AFM analysis confirmed the characteristic surface smoothness and also portrayed the 3-dimensional structure of the nanoparticles.  Figure 4 shows the average size of 20.65 nm of copper sulfide nanoparticles. The smooth-surfaced and spherical-shaped nanoparticles were observed from AFM. Scanning electron microscopy analyzed the size and shape of the synthesized CuS nanoparticles, which are shown in Figure 5. It reveals that the copper sulfide nanoparticles were in spherical and rod shaped with the size range of 20 to 200 nm. EDX graph shows the elemental composition of biosynthesized CuS nanoparticles (Figure 6). A strong signal was received at 1 keV assigned to elemental copper, and a weak signal at 2.3 keV displays the sulfur component. Other peaks like carbon, oxygen, and sodium are associated with CuS nanoparticles.

### 4.4. Biomedical Applications

#### 4.4.1. Antibacterial Activity

Antibacterial activity of copper sulfide nanoparticles was performed against gram-positive and gram-negative bacteria. The bacterial pathogens, namely, *E. coli*, *Vibrio harveyi*, *Vibrio parahaemolyticus*, *Bacillus* sp., and *Proteus* sp., were used in this study. From the antibacterial assay, CuS nanoparticles exhibited the highest zone of inhibition in *E. coli* with 9.00 ± 0.35 mm at the highest concentration.

**Figure 7:**
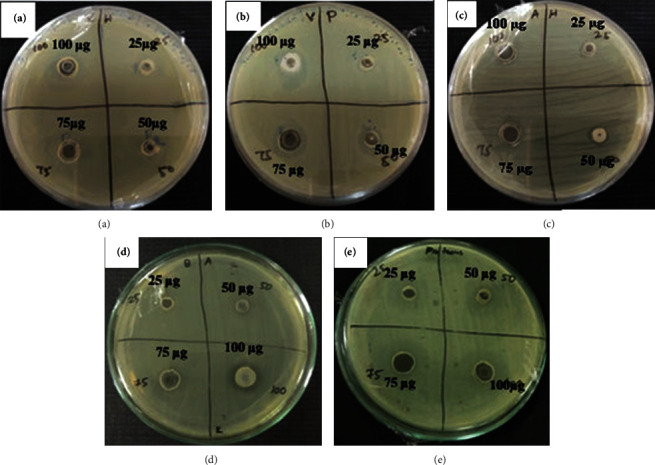
Antibacterial activity of CuS nanoparticles against pathogenic bacteria: (a) *V. harveyi*; (b) *V. parahaemolyticus*; (c) *E. coli*; (d) *Bacillus sp*; (e) *Proteus* sp.

The results obtained for the other three concentrations are 5.31 ± 0.20 mm, 5.99 ± 0.34 mm, and 7.99 ± 0.34 mm for 25, 50, and 75 *μ*g/mL, respectively. Copper sulfide NPs are more active against *Proteus* sp. recorded a maximum zone of inhibition with the size 12.11 ± 0.25 mm in the concentration of 100 *μ*g/mL, which confirmed potential antibacterial activity against *Proteus sp.* than on other tested pathogens. Activity against *Vibrio harveyi* obtained 7.08 ± 0.43 mm, 7.32 ± 0.48 mm, 10.77 ± 0.16 mm, and 11.11 ± 0.28 mm for the tested concentrations. Similarly, activity against *Vibrio parahaemolyticus* was measured as 5.35 ± 0.32 mm, 7.48 ± 0.41 mm, 9.43 ± 0.10 mm, and 12.03 ± 0.07 mm for the tested concentrations, respectively. For *Bacillus* sp., the zone of inhibition was recorded as 5.08 ± 0.37 mm, 5.39 ± 0.21 mm, 9.41 ± 0.32 mm, and 10.58 ± 0.09 mm. The concentration of nanoparticles increases in the treatment against pathogenic bacteria; consequently, the zone of inhibition was increased (Figure 7).

#### 4.4.2. Antioxidant Activity

**Figure 8:**
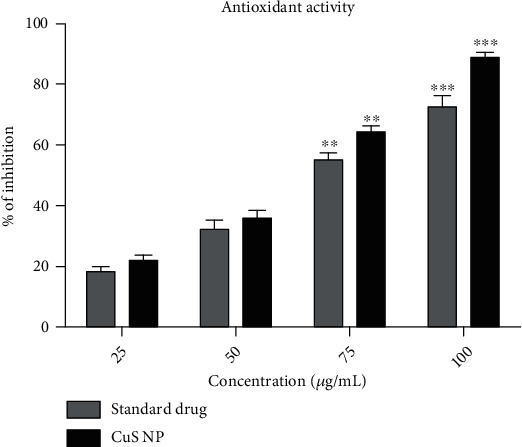
Antioxidant activity of copper sulfide nanoparticles. The error bar values are expressed as mean ± SE. Significant differences were expressed as *p* < 0.001 (∗∗∗), *p* < 0.01 (∗∗), and *p* < 0.05 (∗), and all the experiments were performed in triplicate.

Antioxidant activity of synthesized copper sulfide nanoparticles by DPPH free radical scavenging assay showed increased activity, while increasing the concentrations. The highest inhibition was exhibited by CuS nanoparticles at the maximum concentrations (100 *μ*g/mL). The result observed that increasing the concentration of CuS nanoparticles increases the percentage of antioxidant activity (Figure 8). Statistically, from the two-way ANOVA test, the experimental *p* value recorded here is less than 0.05. In this connection, copper sulfide nanoparticles caused a significant increase in the antioxidant activity at the concentrations of 75 *μ*g/mL and 100 *μ*g/mL, when compared to the standard drug, ascorbic acid. DPPH reduction activity of nanoparticles showed the percentage of inhibition as 64.37 ± 1.11%, which is higher than the standard drug (55.16 ± 1.28%) at the concentration of 75 *μ*g/mL (*p* < 0.01). Whereas at 100 *μ*g/mL concentration, copper sulfide nanoparticles exhibited the most significant (*p* < 0.001) increase in the antioxidant activity (88.71 ± 0.98%) than the standard drug (72.50 ± 2.21%). Other concentrations (25 *μ*g/mL and 50 *μ*g/mL) did not show any significant differences between CuS nanoparticles and standard drugs. IC_50_ values of nanoparticles and commercial drug that required for 50% reduction of DPPH radical were found to be 55.43 ± 0.33 and 67.25 ± 1.62 *μ*g/mL, respectively.

#### 4.4.3. Anti-Inflammatory Activity

**Figure 9:**
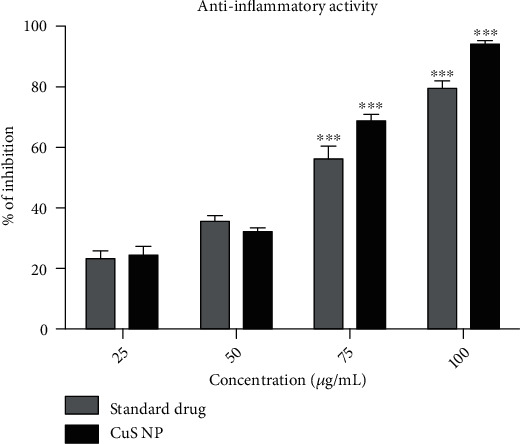
Anti-inflammatory activity of synthesized copper sulfide nanoparticles. The error bar values are expressed as mean ± SE. Significant differences were expressed as *p* < 0.001 (∗∗∗), *p* < 0.01 (∗∗), and *p* < 0.05 (∗), and all the experiments were performed in triplicate.

Anti-inflammatory activity was carried out by the membrane-stabilizing method, and the OD value of inhibition was recorded at 565 nm. The percentage of anti-inflammatory activity was calculated, and the graph of copper sulfide nanoparticles shows the maximum inhibition at 100 *μ*g/mL concentration when compared to the other concentrations. The results have shown that CuS nanoparticles actively inhibited the heat-induced hemolysis. Statistically, the two-way ANOVA shows the experimental *p* value is less than 0.05. Therefore, it was clear that the copper sulfide nanoparticles shows a significant increase in the anti-inflammatory activity at the increasing concentrations compared to the standard drug. The percentage of inhibition produced by CuS nanoparticles was found to be 68.68 ± 1.29%, which is highly significant than standard drug (56.25 ± 2.34%) at the concentration of 75 *μ*g/mL (*p* < 0.001). At 100 *μ*g/mL concentration, the nanoparticles exhibited the most significant increase (*p* < 0.001) in the activity (93.87 ± 0.80%) than standard drug (79.55 ± 1.32%). Other concentrations did not show any significant differences between nanoparticles and standard drug. The inhibition of hemolysis by the nanoparticles at 50% inhibition concentration (IC_50_) was calculated and compared with the standard drug. The IC_50_ values for the CuS nanoparticles and standard drugs were recorded as 47.23 ± 0.33 and 62.72 ± 0.45 *μ*g/mL, respectively (Figure 9).

### 4.5. Toxicology Study of Copper Nanoparticles on the Zebrafish Model

**Figure 10:**
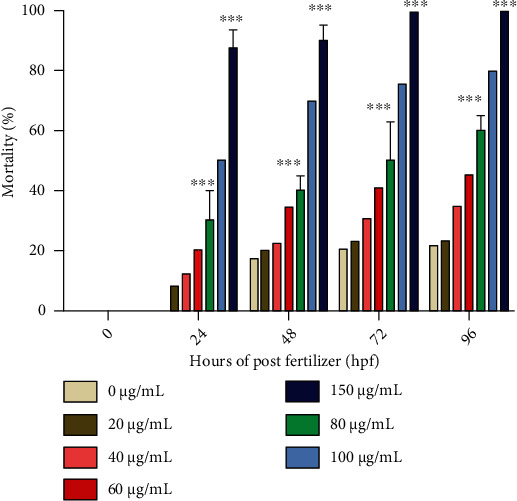
Mortality rate of zebrafish embryos treated with copper sulfide nanoparticles. The error bar values are expressed as mean ± SE. Significant differences were expressed as *p* < 0.001 (∗∗∗), *p* < 0.01 (∗∗), and *p* < 0.05 (∗), and all the experiments were performed in triplicate.

Embryos prior to 4 hpf (sphere stage) were treated with different concentrations of copper sulfide nanoparticles, and developmental abnormalities were observed in embryos treated at different concentrations (20-150 *μ*g/mL).  Figure 10 represents the toxicity of untreated and nanoparticle-treated groups, which shows no significant (*p* > 0.05) effects at the concentrations of 20 and 60 *μ*g/mL, respectively, at all the exposure times. The increased concentrations of 80 and 150 *μ*g/mL of CuS nanoparticles caused highly significant effects (*p* < 0.001) on mortality in embryos. The mortality rate of the fish was gradually and significantly (*p* < 0.05) increased up to 96 hpf while increasing the concentration of nanoparticles. Both 100 and 150 *μ*g/mL of CuS nanoparticles caused 80 and 100% mortality off the embryos, respectively, at the period of 96 hpf. There is a significant effect noticed in 150 *μ*g/mL at the exposure time of 72 and 96 hpf of the embryos. Noticeably, the 50% of lethal concentration (LC_50_) of CuS nanoparticles was found to be 60 *μ*g/mL.

**Figure 11:**
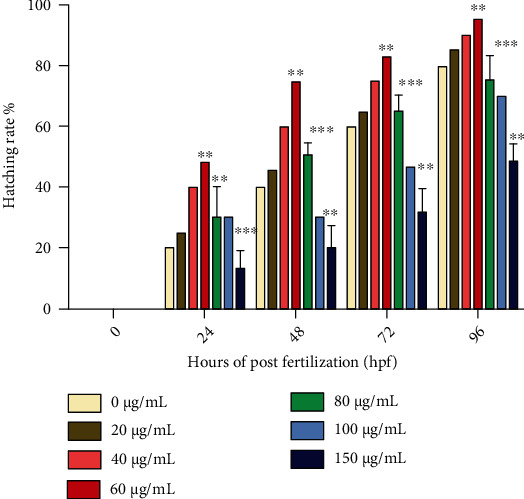
Hatching rate of zebrafish embryos using copper sulfide nanoparticles. The error bar values are expressed as mean ± SE. Significant differences were expressed as *p* < 0.001 (∗∗∗), *p* < 0.01 (∗∗), and *p* < 0.05 (∗), and all the experiments were performed in triplicate.

Likewise, the hatching rate of zebrafish embryos is also affected by the increasing concentration of CuS nanoparticles. The untreated embryos have shown an 80 ± 2.9% hatching rate, whereas 60 *μ*g/mL CuS nanoparticle-treated embryos showed 95 ± 1.7% hatching rates, respectively. There are no significant differences observed in the hatching percentage at 20 and 40 *μ*g/mL copper sulfide nanoparticle-treated groups. The concentration of 40 and 60 *μ*g/mL exhibits *p* value as less than 0.01 (*p* < 0.01). This hatching rate was found to be moderately significant (*p* < 0.05) and decreased while increasing the concentration of nanoparticles up to 150 *μ*g/mL.  Figure 11 shows significantly delayed hatching ability while increasing the concentration of CuS-NPs. Profoundly, the higher concentration of CuS nanoparticles caused developmental toxicity and growth retardation in zebrafish.

**Figure 12:**
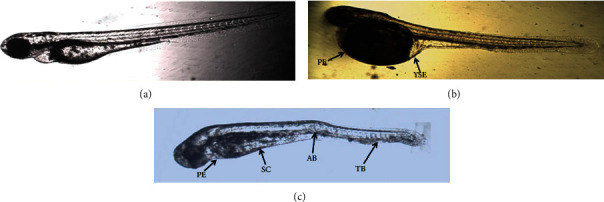
Microscopic image of the zebrafish embryo recorded 80 *μ*g/mL of CuS nanoparticles at different time periods: (a) 24 hpf: not shown toxicity; (b) 48 hpf: shows PE (precardial edema) and YSE (yolk sac edema); (c) 96 hpf: shows SC (spinal cord curvature), AB (axial bent), and TB (tail bent).

The untreated control zebrafish shows average growth without any delayed activity. The 60 *μ*g/mL concentration did not show any significant malfunctions or developmental toxicity up to 96 hpf. Above 80 *μ*g/mL of CuS nanoparticles caused tail and spinal cord flexure and truncation, yolk sac edema, and fin abnormalities. Axial bent and tail bend, pericardial edema was identified through a microscope, which was indicated by arrows, head and eye hypoplasia, and no swim bladder and reduced digestive gut were observed in CuS-NP-exposed embryos (Figure 12). The abnormality was observed in 80 *μ*g/mL CuS-NP-treated groups at 24 hpf, 48 hpf, and 96 hpf.

## 5. Discussion

The formation of greenish-brown indicates the reduction of Cu^2+^ to zerovalent copper (Cu^0^) in the reaction mixture. The mechanism behind this reduction is copper sulfate which is dissociating into Cu^2+^ and sulfate initially. Further, the Cu^2+^ is reduced into zerovalent copper sulfide nanoparticles (Cu^0^) through the biomolecules present in the culture supernatant [30]. Similarly, Pradhan et al. [31] have observed blue color, and later, it could be changed into brown by the involvement of lemon extract.

UV-vis spectrum showed the highest peak at 307 nm, which could be due to surface plasmon resonance effects of copper sulfide nanoparticles [32]. The peak intensity was increased by increasing the time of incubation of reaction mixture. The present results were in good accordance with Rawat et al. [17] who observed the range from 220 to 380 nm for copper nanoparticles during UV-vis spectrum analysis. Presently, some minor peaks were observed as fluctuations, which corresponded to the biomolecules associated with cell-free supernatant that may not be actively involved in the synthesis [33].

XRD spectrum shows two distinct peaks at the 2*θ* values, 31.18° and 44.81°, which are corresponding to the respective (hkl) planes of (110) and (111). These diffraction peaks are well-matched with the pattern of the FCC (face-centred cubic) phase of copper sulfide nanoparticles (JCPDS 04-0836). The average size of nanoparticles observed in this present study (20 nm) using the Debye-Scherrer equation was found to be proficient due to its size which implies more surface area to volume ratio might act as a good candidate to target drug delivery. Also, the present findings are in line with Rosy et al. [34] who have synthesized that the copper nanoparticles in 56 ± 8 nm sized from *Cissus arnotiana* proved significant biological properties.

The functional groups associated with the biosynthesized copper sulfide nanoparticles using cell-free supernatant of *A. hydrophila* were characterized. It was clear that the functional groups, N-O asymmetric stretch nitro compounds, and alcohols involved in the synthesis have been confirmed in the dried and purified copper nanoparticles by comparing the earlier results of Rasouli et al. [35] who have stated that carboxylic acid from protein molecules in supernatant might be responsible for the synthesized nanoparticles. The absorption peak at 1627 cm^−1^ corresponds to protein linkages that interact with nanoparticles and reduce copper ions (Cu^2+^) to copper nanoparticles (Cu^0^) which have been well coincided with Patel et al. [36], who reported the exact wavenumber in their copper nanoparticle biosynthesis.

AFM study revealed the surface characters of synthesized nanoparticles which are showing the rough and smooth surfaces. The average size of CuS nanoparticles is 20.65 nm, which is approximately equal to the XRD size calculation. The presently obtained sizes of the nanoparticles are in accordance with the earlier findings of Karthik and Singh [37] who reported the average size of nanocopper from AFM images as 6.45 nm.

The morphological characters like size and shape of nanoparticles were determined using SEM. The biosynthesized copper sulfide nanoparticles are homogenous and uniformly dispersed. Some are observed as rod and spherical with the size ranging from 20 to 200 nm. Copper and sulfide were bonded to each other, and it appears clustered. Similarly, Ghidan et al. [38] and Khani et al. [39] reported the average size observed between 10 and 100 nm using *Punica granatum* and 5–20 nm using *Z. spina-christi* supernatants', respectively. Inspiringly, the CuS-NPs may elicit a good biological activity due to their different morphological characteristics.

EDX analysis showed the presence of different elemental composition of biosynthesized CuS nanoparticles. The EDAX pattern confirmed respective peaks for copper and the sulfur component as well as the other peaks called biomoities, which may be evolved from the culture supernatant of *A. hydrophila* [40].

Copper sulfide nanoparticles exhibited the preponderant inhibition activity against gram-negative pathogenic bacteria like *E. coli*, *Vibrio harveyi*, *Vibrio parahaemolyticus*, and *Proteus* sp. The gram-positive *Bacillus* sp. resulted in a low inhibition activity that might be due to integrity of cell wall where evasion of the nanoparticles is quite difficult, as they have thick and strong cell wall composition, and made up of complex peptidoglycan [41]. Whereas in the gram-negative bacteria, the nanoparticles may easily penetrate the cell and cause leakage of cell components. Sometimes, nanoparticles were altering either DNA or RNA and even thus lead to cell death [41, 42].

Free radicals are highly uncharged and unstable molecules that contain one or more unpaired electrons which are highly reactive to produce any toxic components. *In vitro* radical scavenging activity of biosynthesized copper sulfide nanoparticles has been evaluated against DPPH. Biosynthesized CuS nanoparticles exhibited significant scavenging activity when compared to the standard drug, ascorbic acid. This difference could be achieved by NPs due to the presence of carboxylic acid, alkanes, and alcohol. *In vitro* anti-inflammatory activity (inflammation inhibition) of biosynthesized CuS nanoparticles was determined by the membrane stabilizing method. The nanoparticles exhibited excellent percentage of inhibition around 93.71%, when compared to a standard drug (79.43%). Comparably, the presently recorded inhibition is prominent as like the previous reports of Tiwari et al. [43] who obtained 92% of inflammation inhibition from the biosynthesized copper nanoparticles.

Toxicity assay of CuS nanoparticles carried out on zebrafish embryos at different concentrations and exposure time. The mortality rate and hatching rate were affected with the increased concentration of nanoparticles. Biosynthesized CuS nanoparticles were found to be toxic to the zebrafish that resulted above 80 *μ*g/mL. Diplomatically to the above biological activity, though the CuS-NPs are potential enough, presently, the nontargeted toxic potentials have not been favored in the acceptable dose of copper nanoparticles in various biomedical applications, i.e., below 80 *μ*g/mL. On contrary to this, various abnormalities like axial bent, spinal cord curvature, tail bent, and yolk sac edema were observed at 80 *μ*g/mL concentration of CuS-NPs. Similarly, Rajendran et al. [44] reported the toxic effect of zirconium nanoparticles on zebrafish. They obtained LC_50_ values for Zr nanoparticles to be 1 *μ*g/mL. Then, 2 to 5 *μ*g/mL shows delayed hatching ability and increased concentration caused malfunctions and developmental retardation in zebrafish.

## 6. Conclusion

One of the affordable and reproducible sources for biosynthesis, the bacteria (*A. hydrophila*), a very perceptible method compared to physical- or chemical-based ones for synthesizing metal nanoparticles was successfully achieved. Bacteria-mediated synthesis can also save time, can be a cost-effective one, and can be grown large scale at an optimum condition. Hence, bacteria will be a best choice as an impending source for the improvement in nanotechnology to synthesize nanoparticles for large-scale production. Extracellular synthesis using *Aeromonas hydrophila* and its biomedical applications have been extensively studied in this present research. Nanoparticle synthesis was characterized and confirmed by XRD, FTIR, UV, SEM, and AFM analyses. Applications such as antibacterial, anti-inflammatory, and antioxidant activity and the nontargeted toxicology effects of copper nanoparticles using zebrafish were also been evaluated. Future studies are warranted with further characterization and field applications.

## Data Availability

The data used to support the findings of this study are included within the article.
